# Two different hematocrit detection methods: Different methods, different results?

**DOI:** 10.1186/1756-0500-3-65

**Published:** 2010-03-09

**Authors:** Marco Bosshart, John F Stover, Reto Stocker, Lars M Asmis, Jörg Feige, Thomas A Neff, Reto A Schuepbach, Silvia R Cottini, Markus Béchir

**Affiliations:** 1 Surgical Intensive Care Medicine, University Hospital of Zurich, CH 8091 Zurich, Switzerland; 2 Department of Internal Medicine, Division of Hematology, University Hospital of Zurich, CH 8091 Zurich, Switzerland

## Abstract

**Background:**

Less is known about the influence of hematocrit detection methodology on transfusion triggers. Therefore, the aim of the present study was to compare two different hematocrit-assessing methods. In a total of 50 critically ill patients hematocrit was analyzed using (1) blood gas analyzer (ABLflex 800) and (2) the central laboratory method (ADVIA^® ^2120) and compared.

**Findings:**

Bland-Altman analysis for repeated measurements showed a good correlation with a bias of +1.39% and 2 SD of ± 3.12%. The 24%-hematocrit-group showed a correlation of r^2 ^= 0.87. With a kappa of 0.56, 22.7% of the cases would have been transfused differently. In the-28%-hematocrit group with a similar correlation (r^2 ^= 0.8) and a kappa of 0.58, 21% of the cases would have been transfused differently.

**Conclusions:**

Despite a good agreement between the two methods used to determine hematocrit in clinical routine, the calculated difference of 1.4% might substantially influence transfusion triggers depending on the employed method.

## Findings

Measurement of hemoglobin or hematocrit is of major importance in contemporary patient care. Anemia or traumatic/surgical blood loss might require transfusion of red blood cells (RBC) to increase oxygen delivery and counteract tissue hypoxia. However, clear transfusion triggers are difficult to define because there is no clear delineation between risk and benefit. To date, at least 3 large randomized controlled trials have compared restrictive transfusion triggers to a more liberal transfusion regime in critically ill adult patients[1], pediatric intensive care patients[2] and premature infants [3]. All three studies showed that a restrictive transfusion regime reduced RBC transfusion requirements without increasing morbidity and mortality. In this context, hemoglobin concentrations ranging from 7-9 g/dl were compared to 10-12 g/dl.

In this context, we have observed a discrepancy between the hematocrit values determined by routine arterial blood gas analysis compared to routine analysis in our central laboratory. Therefore, we analyzed 250 blood samples taken from 50 critically ill patients. The aims were (1) to compare two different hematocrit-testing methods routinely used in our intensive care unit (ICU) and hospital (central laboratory) and (2) to determine whether these two methods might provide different transfusion triggers.

In accordance with the principles outlined by the World Medical Association declaration of Helsinki and following approval by the local Ethics Committee which waived the need for written informed consent for this post hoc data analysis, patient data from 50 patients treated on our intensive care unit from November 2007 to March 2008 were analyzed retrospectively. A total of 50 critically ill patients treated consecutively in our department were included irrespective of hemodynamic stability or diagnosis for the comparison of the two methods.

Blood was drawn daily, usually, at 6 a.m. for routine laboratory analysis, including 2 hematograms performed with 2 different methods. To compare hematocrit values determined by these two different methods of analysis, 250 paired hematocrit values (data of 50 patients over 5 consecutive days resulting in 250 paired samples) were considered. The patients were assigned to 2 different groups depending on the transfusion targets set during ICU treatment. A total of 20 patients with severe traumatic brain injury, severe burns, following reconstructive surgery or with cardiac ischemia had a hematocrit target of 28%. The other 30 patients had a target hematocrit of 24%.

On the ICU arterial blood samples were routinely drawn in 4 to 6 hour intervals to control paO_2_, paCO_2_, hematocrit, glucose or potassium serum levels, required to optimize ventilatory settings, guide transfusions, and adapt intravenous infusion of insulin or potassium. These blood samples were analysed by the nurses using the commercially available blood gas analyzer ABLflex 800 (Radiometer Medical, Copenhagen, Denmark; http://www.Radiometer.com) located on our ICU. Blood was drawn daily, usually, at 6 a.m. for routine laboratory analysis. Among others, differential blood count including analysis of hemoglobin/hematocrit was performed. As a standardized pre- analytic procedure[4,5], at least 2 ml of blood were discarded to prevent hemodilution by infused fluids before withdrawing blood for the actual analysis using specialized syringes, i.e., the syringe for the ABLflex 800 (Arterial Blood sampler 1.7 ml, Radiometer Copenhagen, Denmark) and vacutainers (BD Vacutainer^® ^K2E 5.4 mg, BD-Plymouth, PL67BP, UK) for the ADVIA 2120.

### Method 1: ABLflex 800

The specimen is drawn from the syringe into the cuvette within the gas analyzer maintained at 37°C. Thereafter, one microliter of the specimen is hemolized via ultrasound (30 kHz). Hemoglobin content is assessed spectrophotometrically using 128 different wave lengths (478 to 672 nm). The light is transmitted via glass-fiber optics through a diffraction gating, which diffracts the light into 128 single wavelengths. The detecting device consists of 128 photo diodes. According to the equation of Lambert-Beer hemoglobin content (ctHb) of the blood sample is determined. Based on the ctHb the hematocrit (hct) is calculated via an internal algorithm.

### Method 2: ADVIA 2120

(Siemens Medical Solutions Diagnostics, Zurich, Switzerland, http://www.diagnostics.siemens.com): This device uses 2 sequential methods of haemoglobin measurement:

(1) Flow cytometry: In a first step red blood cells are applied to iso-volumetric sphering and partial fixation. Then a second reagent encases the sample stream and the entire specimen passes through a flow cell. A red laser then measured cell volume and intracellular haemoglobin concentration. The amount of light scattered at low angle (2-3°) is dependent on the cell volume and the high angle (5-15°) is related to the refractive index of the cell, reflecting the haemoglobin concentration for red blood cells (CHCM). The haematocrit then is calculated.

(2) A cyanide-free reagent (borate solution) and a surfactant (N, N,-dimethyllaurylamine N-oxide) cause haemolysis and facilitate the oxidation of haeme iron to Fe 3^+^. Because of the alkaline pH of the reagent haemoglobin loses most of its salt bridges. The ligated heme groups are solubilized by surfactant micelles to generate a green end-product which can be detected photometrically[6].

During analysis both values for haemoglobin determined by 1 and 2 are compared by an internal algorithm. In case of a predefined discrepancy the haemoglobin value is corrected in respect to the colorimetric method which is considered as the "gold standard" [7].

Our null hypothesis was that the two different methods (ABLflex 800 and ADVIA 2120) were equal and did not provide different transfusion triggers. The alternative hypothesis was that the ABLflex 800 method is not equal to the ADVIA 2120 method.

Bland-Altman analysis corrected for repeated measurements [8,9] and Cohen's kappa statistics [10] were used to compare the two methods (Statview 4.5, abacus concepts, Berkeley, CA, USA). Cohen's kappa statistics compares the observations of two different methods and therefore results in 4 possible cases: +/+, +/-, -/+ and -/-. There is an agreement of the methods in the cases of +/+ and -/- (i.e. "transfused"/"transfused" and "not transfused"/"not transfused") and there is no agreement in the cases +/- and -/+ (i.e.,"transfused"/"not transfused" and "not transfused"/"transfused"), respectively. With kappa ≤ 0.4 the agreement is poor, if kappa is between 0.4 and 0.75 agreement is fair and kappa ≥ 0.75 reflects excellent agreement between these two methods.

Comparisons between groups were calculated either with Chi-square test, with a nonparametric Mann-Whitney test or ANOVA log rank test (Statview 4.5, abacus concepts, Berkeley, CA, USA). Statistical significance level was accepted with p < 0.05.

The investigated 50 patients were grouped according to the pre- defined transfusion triggers (24 and 28%) used on our intensive care unit for different illnesses, resulting in 30 patients within the 24-%-hematocrit-group and 20 patients in the 28-%-hematocrit-group. As depicted in table 1 these two hematocrit target groups consist of heterogeneous patients with different leading diagnosis which, in turn, dictated different treatment concepts and different transfusion triggers. Consequently, these 2 groups are not comparable. Serum levels of lactate and body temperature were significantly different in the 2 groups (p = 0.02 and 0.04, respectively). Hospital mortality was also significantly different (p = 0.03).

**Table 1:** Baseline and clinical characteristics

Parameter	24%-hct-group	28%-hct-group	p-value
Number of patients	30	20	
Men	21 (70%)	15 (75%)	0.70
Women	9 (30%)	5 (25%)	0.70
Age (yrs.)	52.5 ± 13.6	44.8 ± 28	0.17
Heart rate (bpm)	94 ± 24	83 ± 22	0.07
MAP (mmHg)	76 ± 15	83 ± 11	0.11
Weight (kg)	87.2 ± 34.7	70.8 ± 11.4	0.10
Height (cm)	172 ± 10	174 ± 10	0.70

Diagnosis			
Sepsis	11 (36.7%)	0	
Lung-TPL	3 (10%)	0	
Liver-TPL	2 (6.7%)	0	
Fascitis	1 (3.3%)	0	
Mesothelioma	1 (3.3%)	0	
Liver Cirrosis	1 (3.3%)	0	
Colon-Carcinoma	1 (3.3%)	0	
Severe Brain injury	0	9 (45%)	
Polytrauma	8 (26.7%)	2 (10%)	
Severe burn injury	0	8 (40%)	
ARDS	1 (3.3%)	0	
Gastric bypass	1 (3.3%)	0	
Cardiac arrest	0	1 (5%)	

Lactat (mmol/l)	1.7 ± 1.8	1.2 ± 0.7	0.02
Temp. (°C)	37.0 ± 1.5	36.7 ± 1.3	0.04
Blood glucose (mmol/l)	6.7 ± 1.3	6.7 ± 1.9	0.15
Norepinephrine (microg/min)	7.9 ± 11.5	8.3 ± 12.8	0.10

SAPS II	39 ± 17	31 ± 15	0.07
Hospital mortality	15 (50%)	4 (20%)	0.03

**Figure 1:**
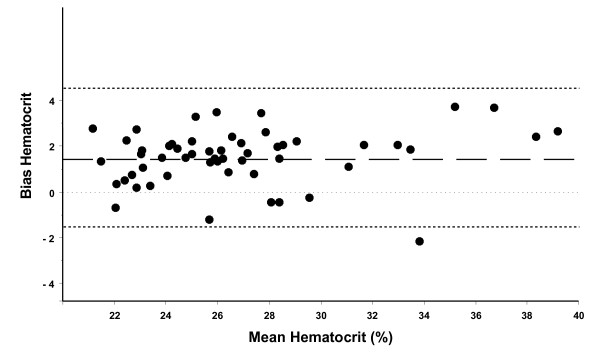
**Bland-Altman analysis for repeated measurements of all patients showed a good agreement with a bias of +1.39% and 2 standard deviations (2 SD) of ± 3.12%**. The lower and upper limits of agreement (bias ± 2 SD) are -1.73 and 4.51%, respectively. Thus, the ABLflex 800 method showed elevated hematocrit by approximately 1.4%.

Bland-Altman analysis for repeated measurements of all patients showed a good agreement with a bias of +1.39% and 2 standard deviations (2 SD) of ± 3.12% (figure 1). Thus, the ABL800 flex method showed an approximately 1.4% higher hematocrit than the ADVIA 2120 method. Bias for each group was not statistically different (1.11 ± 1.83% in the 24%-hematocrit-group vs. 1.70 ± 1.24% in the 28%-hematocrit-group, p = 0.26).

**Figure 2:**
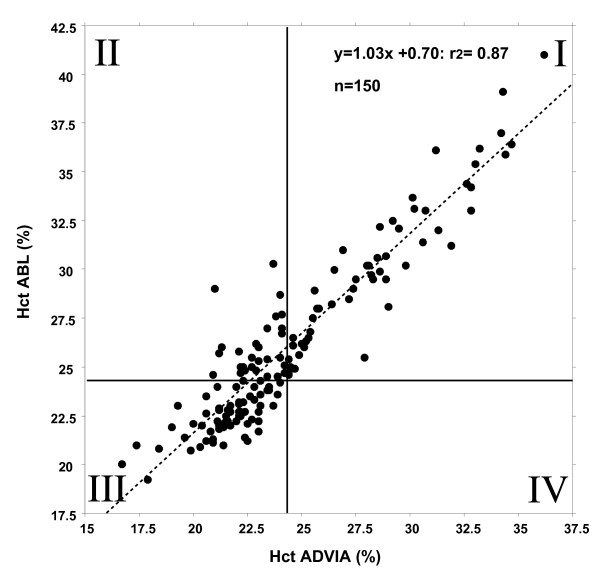
**In 30 patients with a predefined hematocrit target of 24% (n = 150 values) both methods showed a significant correlation of r^2 ^= 0.87 and kappa was 0.56, indicating a fair agreement of the methods**. Values located within the lower field below 24% (square III) reflect patients who need to be transfused according to both methods of analysis; in contrast, square II shows 34 patients (22.7%) who would have been transfused according to the hematocrit values determined by the central laboratory.

According to the targeted transfusion triggers of 24% and 28% two kappa statistical analysis were performed in 30 patients with a transfusion trigger set at hematocrit of 24% and 20 patients with a hematocrit transfusion trigger of 28%. In those patients with a predefined hematocrit target of 24% (n = 150 values) both methods showed a significant correlation of r^2 ^= 0.87 (figure 2). Kappa of 0.56 indicated a fairly good agreement of the methods, i.e., values located within the lower field below 24% (square III) reflect patients who needed to be transfused according to both methods of analysis and square I depicts those patients in whom transfusions were not required, regardless of analytical procedure. In contrast, square II shows 34 patients (22.7%) who would have been transfused according to the hematocrit values determined by the central laboratory. According to the blood gas analysis, however, these patients would not have been transfused.

**Figure 3:**
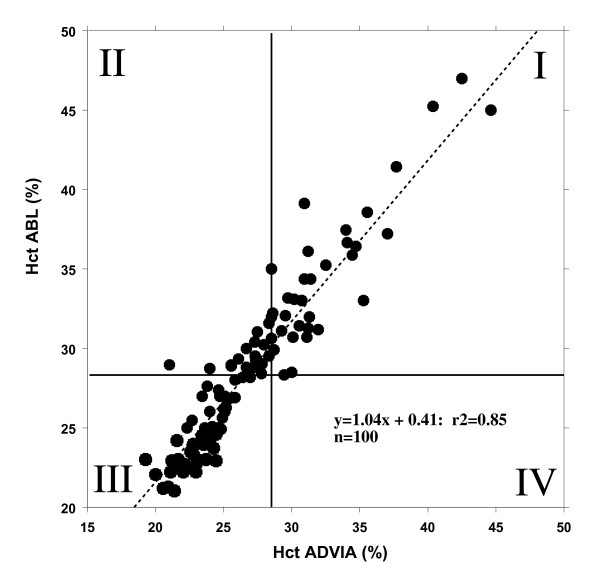
**In 20 patients with predefined hematocrit target of 28% (n = 100 values) both methods correlated well with r^2 ^= 0.8, a kappa of 0.58 reflected a fair agreement of the methods**. Values located within square III below 28% reflect patients who require RBC transfusions according to both methods of analysis, whereas square II shows 21 patients (21%) in whom hematocrit determined by the central laboratory would have suggested RBC transfusion while blood gas analysis would not have indicated RBC transfusion.

In patients with predefined hematocrit target of 28% (n = 100 values) both methods also correlated well with r^2 ^= 0.8 (figure 3). Kappa of 0.58 also reflected a fairly good agreement of these two methods, i.e., values located within square III below 28% reflected patients requiring RBC transfusions according to both methods of analysis. Square II shows 21 patients (21%) in whom hematocrit determined by the central laboratory would have suggested RBC transfusion while blood gas analysis would not have indicated RBC transfusion. In square I none of the patients would be transfused, regardless of analytical procedure.

According to the present analysis, ADVIA 2120 and ABLflex 800 provide similar hematocrit values. Nevertheless, values close to the lower transfusion/hematocrit threshold will be strongly influenced by the employed analytical method. Thus, these methodological differences must be considered in addition to the defined transfusion trigger to standardize transfusion practice within the individual ICU/hospital and across different hospitals in multi-center trials [1]^-^[3]. In this context, it is of critical importance to use the same analytical procedure.

Analytical procedures are strongly dependent on *in vivo *and *in vitro *influences: In this context, the preanalytic phase is crucial for the subsequent analytic process[11] and most importantly for the interpretation of the obtained results. Point-of-care testing (POCT) has been introduced in clinical routine to provide analytical results more rapidly and to allow shorter therapeutic response intervals. For POCT systems like the ABLflex 800, preanalytic steps are decisive which include correct blood withdrawal with discarding of the first sample and immediate analysis to prevent hemodilution and stability of the specimens, respectively. Inappropriate handling and prolonged delay will result in sedimentation of RBC and will interfere with subsequent analysis. Details for preanalytics in POCT are described by Hicks et al [12]. Another important factor is the time span from blood withdrawal to their processing within the analyzer to optimize stability of hematological analysis which is time-dependent and hematology analyzer dependent[13]. In our ICU the nursing staff is trained in standardized blood withdrawal and immediate blood gas analysis within minutes after blood collection.

The samples for the ADVIA 2120 are directly sent to the hematology laboratory, marked "emergency" which guarantees immediate processing, providing results within one hour. The pre-analytic process and the rest of the testing in the laboratory is highly standardized. Thus, assuming that pre-analytic errors occurred, we would rather have to consider this a systematic error.

The ADVIA 2120 method may be influenced by hemolytic samples or lipemia leading to falsely elevated hematocrit levels, in contrast cold antibodies can decrease hematocrit. In addition, polyglobulia may affect the measurement [14]. In our population polyglobulia or lipemia was not present; cold antibodies were not searched for routinely.

The International Committee for Standardization in Hematology (ICSH) recommends the cyanmethemoglobin method as a reference method for hemoglobin measurement[7].

Although blood gas analyzers as POCT methods are increasingly used to provide rapid analysis of hematocrit in ICU, emergency departments, and operating theaters, data showing reliability between the different methods is scarce. A Belgian multicenter study compared different POCT methods to standard laboratory analyzer and found differences from 0.6 to 4.1% [15] which was corroborated by two other studies[16,17].

To date, we lack detailed studies addressing the impact of different analytical methods on the transfusion management. Although both tested methods showed a fair to good agreement, approximately 21% of our samples might lead to a different transfusion management. Taken together, apart from clinical parameters transfusion triggers must not only be defined as simple values but must be considered as method-dependent parameters. In the literature none of the landmark papers [1-3] mentioned the hemoglobin analyzing method. This, in turn, could in theory, lead to different results of these studies. Most importantly, multi-center trials must use the same method of analysis to avoid false transfusions.

Concerning the ICSH guidelines which recommend the cyanmethemoglobin method as a standard method, the ADVIA 2120 method might be more appropriate to measure hemoglobin/hematocrit. Nevertheless, transfusing according to the ADVIA 2120 method would increase the transfusion rate in our patients with its additional risks.

## Competing interests

The authors declare that they have no competing interests.

## Authors' contributions

RS and MBo helped to design the study and drafted parts of the manuscript, LMA and RAS collected the data, TAN and SRC drafted parts of the manuscript, JFS and JF performed the statistics and MB designed the study, drafted parts of the manuscript and was the leader of the project.

All authors read and approved the final manuscript.
